# Built Environment and Childhood Weight Status: A Multi-Level Study Using Population-Based Data in the City of Hannover, Germany

**DOI:** 10.3390/ijerph17082694

**Published:** 2020-04-14

**Authors:** Yusheng Zhou, Christoph Buck, Werner Maier, Thomas von Lengerke, Ulla Walter, Maren Dreier

**Affiliations:** 1 Institute of Epidemiology, Social Medicine and Health Systems Research, Hannover Medical School, 30625 Hannover, Germany; Walter.Ulla@mh-hannover.de (U.W.); Dreier.Maren@mh-hannover.de (M.D.); 2 Leibniz Institute for Prevention Research and Epidemiology - BIPS, 28359 Bremen, Germany; buck@leibniz-bips.de; 3 Institute of Health Economics and Health Care Management, Helmholtz Zentrum München—German Research Center for Environmental Health (GmbH), D-85764 Neuherberg, Germany; werner.maier@helmholtz-muenchen.de; 4 Department of Medical Psychology, Hannover Medical School, 30625 Hannover, Germany; lengerke.thomas@mh-hannover.de

**Keywords:** obesity, overweight, childhood, built environment, walkability

## Abstract

In recent years, built environmental characteristics have been linked to childhood overweight, but the results remain inconsistent across studies. The present study examines associations between several built environmental features and body weight status (BMI) z-score among a large sample of preschool children in the city of Hannover, Germany. Walkability (Index), green space availability, and playground availability related to preschool children’s home environments were measured using data from OpenStreetMap (OSM). These built environment characteristics were linked to the data from the 2010–2014 school entry examinations in the Hannover city (*n* = 22,678), and analysed using multilevel linear regression models to examine associations between the built environment features and the BMI z-score of these children (4–8 years old). No significant associations of built environmental factors on children’s BMI were detected, but the effect between green space availability and BMI was modified by the parental educational level. In children with lower compared to higher educated parents, a higher spatial availability of greenspace was significantly associated with reduced body weight. Future research should continue to monitor the disparities in diverse built environment features and how these are related to children’s health.

## 1. Introduction

Childhood overweight and obesity have become a global epidemic in the last decades [1]. In Germany, according to the German Health Interview and Examination Surveys for Children and Adolescents (“Kinder- und Jugendgesundheitssurvey”, KiGGS), 15.4% of children aged 3–17 years were overweight and 5.9% were obese (KiGGS Wave 2, 2014–2017) [2]. Though current trend analysis has shown that prevalence rates of overweight and obesity in children has reached stagnation [3,4], it is important to understand their determinants in order to control the epidemic.

In line with the fact that overweight and obesity are multifactorial in origin, the potential impact of the built environment on children’s health has gained increasing attention [5,6]. Among others, the concept of walkability is applied to explore the ways in which built environment characteristics fail to support walking and consequently, influence on body weight. Duncan et al. [7] found that neighbourhood walkability could significantly impact on children’s physical activity levels. According to Carrol-Scott et al. [8], children living in walkable neighbourhoods with adequate spatial measurements have a lower risk of obesity because these environments promote physical activity behaviours. Kowaleski-Jones et al. [9] have shown that children who live in more walkable neighbourhoods have a lower risk of childhood obesity.

In order to operationalize walkability, two area-based measures have been used. The first type is the Walkability Index, which is designed to reflect various built environment elements by capturing the multiple attributes of a place. Frank et al. published it in 2005, and proposed to measure intersection density, net residential density, retail floor area ratios, and entropy scores within the index [10]. Based on the Walkability Index, a Moveability Index was published by Buck et al. in 2011, which further explores the built environment by using the kernel density estimation method. This method is for smoothing point patterns into a generalized surface by applying a kernel function with specified radius to each point in the data set [11]. The second type is a group of measures which emphasize the distribution of potential destinations. These measures examine that a place is more walkable if more amenities are available within certain area, which could better represent from the pedestrian choice. However, this emphasis can be double-edged, as it may be failing to differentiate between amenities and overlooking various walking purpose [12,13]. Here in our study, we decided to choose the former one which is building a Walkability Index based on data availability.

Other than the walkability itself, features which promote an active lifestyle, like greenspaces, parks, and playgrounds, have also been analysed in most of the current studies [14,15]. Liu et al. found that higher availability of greenspace was associated with a decreased risk of overweight, but only among those in areas with a greater population density [16]. Multiple studies indicated that parks within children’s living environment were neighbourhood predictors of childhood obesity [17,18,19]. However, these relationships were highly dependent on the socioeconomic status (SES) of the child’s parents or neighbourhood [20]. There are likely to be many mediators of the relationship between SES and overweight including barriers associated with willingness, time and opportunities (e.g., within a local neighbourhood) to eat a healthy diet, or take part in physical activity [21,22].

A detailed knowledge of the spatial patterns and influencing factors on area level is required to explore association between the built environment and childhood overweight. Although most studies concerning built environmental factors have been mainly located in the United States [21], there is still a gap in European and German research about the built environment impact [23,24]. For that reason, this study contributes to the existing literature and analyses associations between built environment and preschool children in a major German city. Therefore, the goal of this study is (1) to assess the effect of different features, including walkability, greenspace, and playgrounds, in the association between built environment and weight status of children and (2) to assess whether these associations were moderated by SES or other factors

## 2. Materials and Methods

### 2.1. Study Population and Study Area

This study included preschool children in the city of Hannover, the capital of the federal state Lower Saxony in Germany with about half a million inhabitants. The study population (*n* = 22,678) comprised children at the age from 4 to 8 years old (48.5% girls) registered for school entry within a 5-year period from 2010 to 2014. The data collected as part of the school entrance examination provide information about age, sex, height, weight, and the results of the screening on developmental disorders (linguistic, gross or fine motor, psychological, and emotional). Sociodemographic data were collected from the children’ parents using a German-language questionnaire. This information was voluntary. All data were rendered anonymous and had no identifying information. The school entrance examination was run by the standardized examination programme “SOPHIA” (“Sozialpädiatrisches Programm Hannover—Jugendärztliche Aufgaben”—http://www.sophia-online.org) that includes a documentation and evaluation procedure focused on prerequisites relevant for future school success. Permission of data usage was gained. The available data were processed and aggregated on the level of 51 district areas comprising the total number of the city district of Hannover. The area information was based on the administrative boundaries provided by Statistics Office of State Capital Hannover (“Statistikstelle der Landeshauptstadt Hannover”).

### 2.2. Dependent Variable

Weight and height were objectively measured by the medical staff. The BMI (body mass index) z-score was the dependent variable in the present analyses. Height, weight, sex, and age were used to calculate BMI z-score using the Kromeyer-Hauschild reference [25]. This reference is the national weight status reference for German children based on 17 pooled regional surveys conducted in Germany between 1985 and 1999 that used the sex- and age-specific 90th and 97th percentiles as cutoffs. The weight status was categorized into: normal weight (BMI < 90th percentile), preobesity (90th percentile ≤ BMI ≤ 97th percentile), and obesity (BMI > 97th percentile). Being overweight in this study refers to the status including both preobesity and obesity [25].

There are two reasons why we applied the national reference in our study instead of an international reference. First, most studies in Germany were using this reference, e.g., the report of national survey KiGGS conducted by Robert Koch Institute [2]. Thus, our results are likely to be comparable with the overweight prevalence in German children across different populations and time periods. Evidence has shown that the Kromeyer-Hauschild reference is sufficiently strong for estimating the prevalence of childhood overweight/obesity [26]. Another reason is that using national reference data to categorize BMI is more suitable for diagnosing overweight/obesity than the assessment using international references which has been shown in a systematic review [27].

### 2.3. Individual-Level Independent Factors

The children’s individual characteristics were recorded during the school entrance examination. All the parents answered a series of questions asked by the medical assistant. By asking parents to answer their self-defined home country, the children’ ethnicity was categorized into German children and children with migration background. Parental education status was in line with the International Standard Classification of Education (ISCED) [28]. An educational class index consisting of three educational classes for parents (lower, middle, and higher) was created and evaluated by a points system and added together using two indicators (primary qualification and professional education). The family structure was coded as nuclear family (children living with both parents together) or other (a single-parent family/a blended family). The child’s number of siblings was coded into two categories (one or no siblings and two or more siblings). The birth weight was provided by the interviewed parents and categorized into three groups (high: >4000 g, normal: 2500–4000 g, and low: <2500 g). To consider the effect of childcare service usage, we obtained the length of child day care (nurseries, kindergartens, and other day care facility forms) participation, which was coded as 3 years or more or less than 3 years.

### 2.4. Area-Level Sociodemographic Factors

We considered two aggregated variables (percentage of people with migration background and unemployment rates) on the level of the administrative districts describing the sociodemographic characteristics. First, the proportion of residents with migration background of the area was considered. Different culture, genetic and physiological factors, and ethnic difference might boost up unhealthy weight gain [29]. At a macroscale, migrants tend to be geographically concentrated which provide a supportive environment for the retention of traditional diets and lifestyles [30], meaning that an area with a higher proportion of migrants might provide a different obesogenic environment. Second, unemployment rate of the area is expected to be associated with overweight prevalence through a modifying influence of household income [31]. These two area-level variables were provided by the Statistics Office of State Capital Hannover which annually publishes structural data of the city districts. Here, the years from 2010 to 2014 were selected to match the school entrance examination data.

### 2.5. Area-Level Built Environmental Variables

The built environment variables were assessed using OpenStreetMap (OSM). In this study, the OSM data were collected at OSM Geofabrik (Geofabrik GmbH, Karlsruhe, Germany, http://www.geofabrik.de/). Geofabrik provides preprocessed OSM data for free download by continent and country in shapefile format.

Walkability is measured in this study as an indicator of the neighbourhood’s capacity to support physical activity. Walkability Index has been used in the previous literature to assess walkability [10]. All assessments of the built environment features and spatial analyses were conducted within an open source GIS (Geographical Information Systems) software program—QGIS 3.4.5 LTR (QGIS Development Team (2018). QGIS Geographic Information System. Open Source Geospatial Foundation Project. http://qgis.osgeo.org). The following components of the Walkability Index were assessed: intersection density, residential density, and land use mix. Each component was measured in 51 district areas according to Frank et al. [10] and Dobesova et al. [32] and modified to fit the data in Hannover city. The intersection density was derived from the street network as an indicator of street connectivity and was calculated as the ratio between the numbers of true intersections (three or more legs) to the land area. Residential density was measured using household data published by the statistics bureau of Hannover. Land use mix was estimated by an entropy index indicating the evenness of the distribution of different land uses [10]. We applied entropy measures developed by Lawrence Frank and colleagues with a five-category mix: residential, retail, entertainment, office, and institutional [10]. The Walkability Index was obtained by adding the partial scores of the mean of each mentioned indicator after converting them into z-scores in the following expression:

Walkability = [(z-intersection density) + (z-land use mix) + (z-residential density)]

The original Walkability Index further includes a floor ratio to estimate the retail area [32], which is supposed to facilitate the pedestrian access. Yet, similar to several European walkability studies [23,33], the retail floor ratio was left out in this study because in a European context, it may overestimate the actual retail areas, in contrast to land use patterns in the U.S. European land use is shaped by mixed uses within one building, which are either classified as retail or nonretail, and thereby might lead to biased data [23].

In addition to the walkability characteristics, we included greenspace and playgrounds as built environment determinants. Due to their potential health benefits and strong association to the physical activity of children and adolescents, the spatial availability of greenspace and playgrounds has been a focus of planning and research [16,34]. In this study, we determined the area of playgrounds and the area of greenspace within or intersecting each census block group using GIS and included playground availability and greenspace availability as the main independent variables. Both determinants were enumerated using GIS shapefiles from OSM data and double-checked using resources provided by the municipalities within the study area. The greenspace included multiple OSM land use categories comprising areas of open space for recreation, typically having a seminatural state (e.g., including grassy areas, trees, and bushes). We calculated the percentages of area within or intersecting each census block group as additional built environment variables.

### 2.6. Statistical Analyses

We conducted a multilevel linear regression analysis to achieve the research goals. First, an unconditional model with no predictors was estimated to assess the intraclass correlation in BMI z-score. Then, all individual-level characteristics (i.e., sex and migration background) and area level sociodemographic factors (percentage of people with migration background and unemployment rates) were added as fixed effects (Model 1). Model 1 accounts for all the compositional differences across both individual- and area-level in order to examine the unique contribution of our main independent variables, built environment factors. To examine the unique contribution of the three built environment variables (Walkability Index, availability of playgrounds, and availability of greenspace), these were added to the previous model separately (Models 2–4). In order to assess whether these associations were moderated by SES, we assessed the interaction effect of the parental education level. Model 5 represents the main effect models followed by adding the corresponding interaction term between parental educational level with each built environment factor (Model 5a: parental educational level*Walkability Index, Model 5b: parental educational level*playgrounds availability, and Model 5c: parental educational level*greenspace availability). To better display and explain the interaction term discovered, the interaction effect using the predicted values from Model 5 was plotted in a scatter figure. Additionally, logistic regression models fit for the outcome of overweight prevalence were also examined. BMI z-score higher than 1 was categorized as overweight (overweight refers to the status including both preobesity and obesity). The significance level was defined as α = 0.05. Children’s neighbourhood location (district area) was specified as the random effect of these models. Moreover, we compared the model fit throughout the model-building process by examining the changes in the Akaike information criterion (AIC) which shows the preferred model having the lowest value [35]. All analyses were performed using IBM SPSS Statistics for Windows software, version 25.0 (IBM Corp., Armonk, NY, USA).

## 3. Results

The sample used in this analysis included a total of 22,678 children in 51 administrative areas. Of the participants, 51.5% were boys and 48.5% were girls. The age range of the children is 4–8 years (mean = 5.996 and SD = 0.359). The overall prevalence of overweight (preobesity and obesity) was 9.7%, while the obesity prevalence was 4.1%. Half of the children had a migration background (49.4%). For the family structure, 2.8% of the children came from a single-parent or blended family and 30.5% of them had two or more siblings (Table 1).

**Figure 1:**
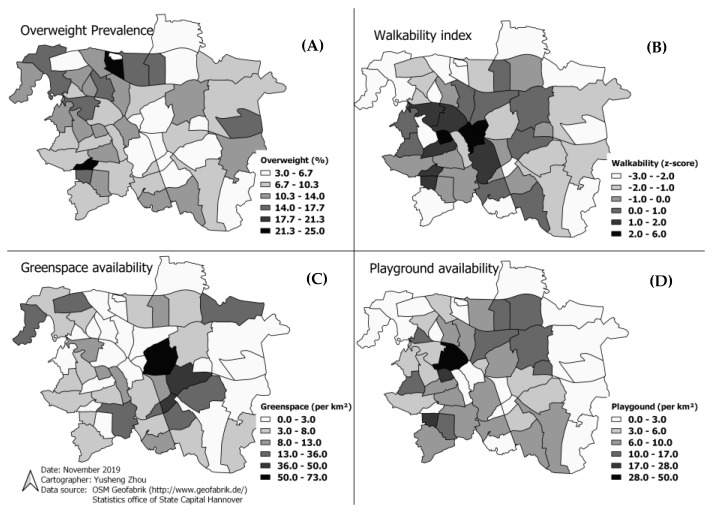
Spatial distribution patterns of overweight in preschool children and built environmental features in the Hannover city ((**A**) Overweight prevalence, (**B**) Walkability Index, (**C**) Greenspace availability, (**D**) Playground availability).

The spatial distribution of childhood overweight in 51 administrative areas in the city of Hannover is shown in Figure 1. A distinct pattern could be identified, with the highest proportion of overweight of more than 14% in the surrounding areas of the city, showing a proportion of more than 14% overweight. In contrast, the inner areas of the city are characterized by low numbers of overweight with less than 5.5%. The distribution of overweight prevalence and the three built environment variables are presented below (Figure 1).

Table 2 presents the results of the multiple linear regression analysis. Across all models, children’s sex, migration background, number of siblings in the family, birth weight, and parental educational level were significantly associated with the children’s weight status. The area-level information (unemployment rate and percentage of residence with migration background) revealed no significant relation with the body weight. Overall, no significant associations were found between each environmental factor and children’s weight status.

Table 3 indicates the interaction term between parental educational levels with each built environment factor. One significant interaction was detected between greenspace availability and parental educational level (Table 3). As seen in Model 5c, the level of parents’ education moderated the association between the greenspace availability and the body weight (*b* = −0.1, 95% CI (−0.19, −0.01)).

**Figure 2:**
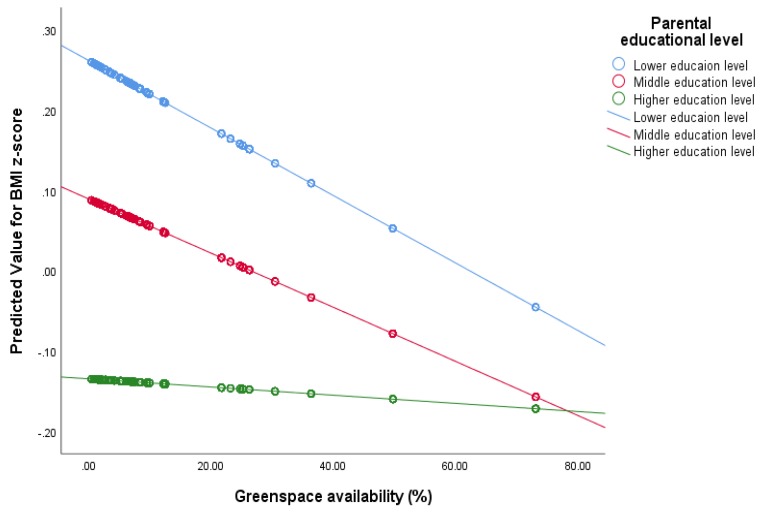
Scatter plot of predicted body mass index (BMI) z-score for children with different parental education level (higher, middle, and lower) by greenspace availability. (Notes: adjusted for sex, migration background, siblings, family structure, child day care participation, birth weight, unemployment rate, and rate of population with migration background per area).

After adjusting for the individual and area-level characteristics, Figure 2 shows that the association of the greenspace availability and children’s weight is almost 0 for the high parental educational level group, while the association of the greenspace and BMI was negative among the children from the low and middle parental educational level groups. That means, a higher availability of greenspace was associated with a lower weight status for children whose parents had lower and middle education levels but not for those whose parents had a higher education level.

Analysis of overweight prevalence using logistic regression yielded similar results. Availability of greenspace was marginally associated with children’s risk of being overweight (OR 0.989, 95% CI 0.985, 0.994). This association was not significant (OR 0.997, 95% CI 0.992, 1.003) after adjustment for other individual and area-level SES characteristics. The Walkability Index and the availability of parks were not significantly associated with children’s overweight prevalence.

## 4. Discussion

In this study, we examined the relationship between overweight in preschool children and several built environmental factors. Our main findings indicated no significant associations between built environmental factors (Walkability Index, availability of playgrounds, and availability of greenspace) and children’s weight status. However, our results suggested an interaction between individual-level SES (parental educational level) and greenspace availability while not for area-level SES: For children with lower educated parents compared with higher educated parents, a higher spatial availability of greenspace was significantly associated with reduced body weight.

Our results resonate with previous findings in the literature suggesting that individual SES factors are strongly associated with childhood BMI. It should be noted that parental educational level is the only available SES factor in our study. Previous studies assessing other SES factors suggest that children from families with low SES are at higher risk of becoming overweight or obese. According to Saelens et al. [36], children from families with low incomes had higher risk of being obese. However, the social gradient in the prevalence of overweight cannot be fully explained by individual factors alone. With the emergence of social ecological theory, the area-level SES has been increasingly investigated as a predictor. Prior studies indicate that adolescents who lived in deprived areas were more likely to be overweight and had higher levels of body fat than adolescents in more affluent areas [8,37]. However, the findings regarding area-level SES are inconsistent. A cross-sectional study found that a disparity in income among families affected the occurrence of childhood obesity, irrespective of neighbourhood SES [38]. In our findings, area-level factors (unemployment rates and percentage of migrants) were not significantly associated with the children’s weight status, which could be partially due to the reason that, at present, there is no universal area effect on health outcome across all population groups. In addition, sex and ethnic differences in weight status were observed in our study. The prevalence of overweight among girls was slightly higher than among boys (10.0% compared to 9.5%), while children with a migration background had a higher overweight risk (13.2% compared to 6.4%). These differences are consistent with previous studies [3,29] and may be due to genetic factors as well as cultural habits. Further studies should consider how the built environment–overweight association may vary by sex and race/ethnicity [15].

No association was found between factors of the built environment, such as greenspace availability, playground availability or walkability, and overweight and obesity on the aggregated level of analysis in preschool children in Hannover. Furthermore, similar studies targeting German children and adolescents were unable to identify a significant association [33,39], except for one study based on data from Munich [23]. This study identified that lack of greenspace, low/middle playground space, and low park space were associated with a higher BMI although only in the bivariate analyses [23]. However, the evidence of an association between built environment and physical activity is robust. Buck et al. [40,41] found a strong variation in this association between physical activity and built environment using several variables, including features of the walkability concept and the availability of recreational facilities such as playgrounds and greenspace.

For greenspace availability, specifically, while most studies showed mixed or weak evidence of a relationship between greenspace and BMI, several reports have indicated a positive relationship (i.e., reduced BMI) between greenspace and BMI. Liu et al. [16] found that increased greenspace availability was associated with reduced weight among children living in areas with a high population density, while Petraviciene et al. [42] reported that less greenness exposure was associated with higher probability of being overweight and obese. All these studies highlighted the potential effect of SES on change in weight status. Since more affluent parents tend to live in more salubrious areas, the effect of the environment may be partly driven by the parental SES [42].

Moreover, the environmental context may matter more for those otherwise unable to take advantage of it. An interaction relationship between the SES and the environmental context on the change in children’s weight status was explored in this study. We were able to demonstrate that the associations between the environment and childhood overweight/obesity were moderated by the educational level of the parents. At the same time, two area-level SES variables failed to provide a significant association. In our study, higher neighbourhood greenspace availability was associated with a lower BMI z-score, while the effect was stronger for children growing up in less educated families compared with children from higher educated families. As a frequently used indicator of SES in health behaviour surveys, parental educational level is believed to reflect the health-related lifestyle among parents, which in turn affects their children’s lifestyle [43]. Our results are consistent with the findings of Lovasi et al. [44,45] who found that children in lower income families had a reduced risk of obesity if they lived in an area with a higher density of trees. Less affluent families might be more restricted to their immediate surrounding and thus benefit more from greenspace availability [14,46].

Physical activity is a potential mechanism through which built environments may influence obesity. Among youth, various elements of the built environment have been linked to increased physical activity. Children with access to recreational facilities, usually close to their neighbourhoods, are more active than those without such access [44]. A large body of literature found associations between neighbourhood walkability and physical activity [47,48]. Some studies identified physical activity to be a mediator of the neighbourhood environment–BMI association [49,50].

In addition to the complex mechanism related to physical activity, many other factors could confound the association between built environment and BMI. “Residential self-selection” has been put forward as a potential important confounder of the positive association between walkability and physical activity. Residential self-selection implies that families are likely to select their neighbourhood according to their culture, lifestyle, and personal preferences, and consequently those who are already active or who wish to be active may choose to live in a high-walkable neighbourhood and vice versa [51]. Many studies of physical activity have control for residential self-selection in their analyses, resulting in mixed findings ranging from significant attenuation of the associations to minimal effects on the associations [51,52,53]. Some residents may choose to live in neighbourhoods that support their activity preferences in some cases. In other situations, residents may prefer to live in neighbourhoods with fewer recreational facilities because of low-cost housing [50,52]. Although these analyses assume that children have little choice in their residential location (as it mostly depends on family selection), residential self-selection remains a significant factor [54]. Overall, without including the residential selection factor, the association of built environment features and children’s BMI might be overestimated. Definitive evidence of the presence or absence of residential self-selection confounding awaits further exploration.

The strength of our study is the large sample size (*n* = 22,678), which enabled multilevel analyses in order to explore how the association between neighbourhood environment and childhood overweight and obesity adjusted for several factors and to create maps illustrating the spatial patterns of overweight across the city of Hannover. Moreover, we were able to obtain objective measures assessing the built environment in this study. Built environment features can be collected using either subjective or objective methods [48,55]. Many studies have applied subjective methods, [56,57] placing considerable value on the subjects’ judgment of their own neighbourhood and the factors that contribute to it. Subjective tools can relate to self-reported perceptions of the environment, including self-evaluations of the subjects’ familiarity with the surroundings. However, studies showed a mismatch between objectively and subjectively measured built environment features, suggesting that environmental perceptions are stronger correlates of activity among children than objective measures in specific situations [58]. Future research could consider combining these two measurements in order to produce a more complete perspective.

The current study has several limitations. First, due to the cross-sectional design, causality cannot be attributed to the observed findings. Reverse causality cannot be disregarded, whereby children with elevated symptoms of overweight/obesity perceive their surrounding built environment more negatively and less conducive to walking behaviour. Second, although we captured an important outcome (BMI z-score) objectively for children in the study, several other unmeasured variables, such as physical activity, may be key mediators or confounding factors in the built environment–overweight relationship [59,60]. Objective measuring of physical activity for over 22,000 children is challenging, but additional research should include multiple health behaviours and outcome measures to better explicate the relationship between key environmental features and overweight. At the same time, there is a potential for residual confounding secondary to unmeasured aspects of the area or individual-level SES measures. Many important SES variables from previous studies, including household income, were not included due to data availability. Moreover, we used administrative boundaries as a proxy for the neighbourhood environment, and this may have induced a misclassification. Our environmental measures were conducted at the area level because individual home addresses were not available. Area-level built environment measurement can be coarse, and variation at a finer or coarser scale (zip code and home address) may be critical in affecting physical activity [61]. Hence, we were unable to assess the sensitivity of our results to different spatial scales [61]. The effect of scales on matched exposure–response relationships in the literature about built environment needs further investigation.

## 5. Conclusions

This study examines the associations between built environments and individual BMI z-score in preschool children. The three built environment factors considered were measured at area level and included a Walkability Index, the availability of playgrounds, and the availability of greenspace. The built environmental factors did not show a significant association with children’s weight status, while the proximity of greenspaces may have a small protective effect on children’s overweight that is restricted to children with low-educated parents. These findings demand a more detailed analysis of the built environment–overweight relationship that considers the amount and location of the physical activity of children.

## Figures and Tables

**Table 1:** Descriptive characteristics of the study population and the area (Data from school entrance examination, city of Hannover, 2010–2014, *n* = 22,678.).

Characteristics		*N* (%)	Mean	Standard Deviation	Minimum	Maximum
	Dependent variables					
Body mass index (BMI) z-score			0.06	1.04	−3.96	3.08
	Overweight *	9.7%				
	Obese	4.1%				
	Individual level factors					
Sex	Boys	51.5%				
	Girls	48.5%				
Migration background	Yes	49.4%				
	No	50.6%				
Family structure	Single parent/blended family	2.8%				
	Nuclear family	97.2%				
Siblings	≥2 siblings	30.5%				
	<2 siblings	69.5%				
Child day care participation	<3 years	18.7%				
	≥3 years	81.3%				
Parental educational level	Higher	36.9%				
	Middle	26.2%				
	Lower	36.9%				
Birth weight	High (>4000 g)	10.6%				
	Normal (2500 g–4000 g)	80.1%				
	Low (<2500 g)	6.6%				
	Area level SES factors					
Unemployment rate (%)			8.7	3.2	1.9	16.1
Proportion of residents with migration background in the area (%)			25.6	9.5	6.5	50.3
	Built environment variables (area level)					
Walkability Index			0.52	1.79	−3.25	6.41
Playground availability (area of playgrounds per km^2^)			10.61 × 10^−3^	9.21	0.15 × 10^−3^	50.09 × 10^−3^
Greenspace availability (percentage of area with greenspace)			8.86	11.50	0.51	73.09

* Overweight refers to the status including both preobesity and obesity.

**Table 2:** Associations between children’s body mass index (BMI) z-score and the characteristics of the study population and the area (Data from school entrance examination, city of Hannover, 2010–2014, *n* = 22,678.).

Independent Variables		Model 1		Model 2		Model 3		Model 4	
		β (SE)	95% CI	β (SE)	95% CI	β (SE)	95% CI	β (SE)	95% CI
Individual-level independent factors									
Girls (Ref. boys)		0.92 (0.15) *	(0.63, 1.21)	0.92 (0.15) *	(0.63, 1.2)	0.92 (0.15) *	(0.64, 1.21)	−3.77 (0.52) *	(−4.82, −2.72)
Children with migration background (Ref. German children)		1.86 (0.16) *	(1.55, 2.17)	1.86 (0.16) *	(1.55, 2.18)	1.86 (0.16) *	(1.55, 2.17)	0.88 (0.16) *	(0.58, 1.19)
Single parent/blended family (Ref. nuclear family)		0.81 (0.44)	(−0.05, 1.67)	0.81 (0.44)	(−0.05, 1.67)	0.81 (0.44)	(−0.05, 1.67)	1.87 (0.17)	(1.54, 2.21)
Two or more siblings (Ref. one or none siblings)		0.57 (0.17) *	(0.24, 0.89)	0.57 (0.17) *	(0.24, 0.89)	0.57 (0.17) *	(0.24, 0.89)	0.66 (0.47) *	(−0.26, 1.58)
Childcare less than 3 years (Ref. 3 year or longer)		−0.15 (0.21)	(−0.55, 0.24)	−0.15 (0.21)	(−0.55, 0.24)	−0.15 (0.2)	(−0.54, 0.24)	0.56 (0.18)	(0.22, 0.91)
Parental educational level(Ref. higher education)	Lower education	2.71 (0.19) *	(2.34, 3.08)	2.71 (0.19) *	(2.34, 3.08)	2.71 (0.19) *	(2.34, 3.08)	2.63 (0.21) *	(2.23, 3.02)
	Middle education	1.56 (0.19) *	(1.19, 1.93)	1.56 (0.19) *	(1.19, 1.93)	1.56 (0.19) *	(1.19, 1.93)	1.52 (0.21) *	(1.12, 1.92)
Birth weight(Ref. normal)	High (>4000 g)	3.69 (0.23) *	(3.24, 4.15)	3.69 (0.23) *	(3.24, 4.15)	3.69 (0.23) *	(3.24, 4.15)	3.58 (0.25) *	(3.11, 4.07)
	Low (<2500 g)	−2.51 (0.31) *	(−3.11, −1.93)	−2.51 (0.31) *	(−3.11, −1.93)	−2.51 (0.3) *	(−3.11, −1.93)	−2.33 (0.32) *	(−2.96, −1.71)
Area-level sociodemographic factors									
Unemployment rate		0.21 (0.09) *	(0.01, 0.39)	0.21 (0.11) *	(0.01, 0.41)	0.21 (0.11) *	(0.01, 0.41)	0.19 (0.11) *	(−0.03,0.41)
Rate of population with migration background in the area		−0.02 (0.03)	(−0.08, 0.05)	−0.02 (0.03)	(−0.08, 0.05)	−0.02 (0.03)	(−0.09, 0.05)	−0.01 (0.04)	(−0.09,0.06)
Area-level built environmental variables									
Walkability Index				0.01 (0.08)	(−0.15, 0.16)				
Playground availability						0.01 (0.02)	(−0.04, 0.03)		
Greenspace availability								−0.01 (0.01)	(−0.03, 0.02)
Akaike information criterion (AIC)		135,677.2		135,676.8		135,646.1		119,530.8	

* *p* < 0.05. Model 1: model on children’s BMI z-score adjusted for individual level factors and area level socioeconomic status (SES) factors reported in the table. Model 2: Model 1 plus Walkability Index. Model 3: Model 1 plus playground availability. Model 4: Model 1 plus greenspace availability.

**Table 3:** Associations between children’s BMI z-score and interaction terms of parental educational level (ref. higher education level) and built environment features (Data from school entrance examination, city of Hannover, 2010–2014, *n* = 22,678.).

Interaction Terms	Model 5a		Model 5b		Model 5c	
	β (SE)	95% CI	β (SE)	95% CI	β (SE)	95% CI
Lower education level * Walkability Index	0.25 (0.1)	(−0.05, 0.44)				
Middle education level * Walkability Index	−0.04 (0.1)	(−0.24, 0.17)				
Lower education level * Playground availability			0.03 (0.02)	(−0.01, 0.07)		
Middle education level * Playground availability			−0.02 (0.02)	(−0.06, 0.02)		
Lower education level * Greenspace availability					−0.04 (0.02)	(−0.07, −0.01)
Middle education level * Greenspace availability					−0.02 (0.02)	(−0.05, 0.01)

Included independent variables: Model 5a: parental educational level, Walkability Index, and parental educational level * Walkability Index. Model 5b: parental educational level, playground availability, and parental educational level * playground availability. Model 5c: parental educational level, greenspace availability, and parental educational level * greenspace availability.
